# Bibliometrics of systematic reviews: analysis of citation rates and journal impact factors

**DOI:** 10.1186/2046-4053-2-74

**Published:** 2013-09-12

**Authors:** Pamela Royle, Ngianga-Bakwin Kandala, Katharine Barnard, Norman Waugh

**Affiliations:** 1 Division of Health Sciences, Warwick Medical School, University of Warwick, CV4 7AL, Coventry, UK; 2 University of Oxford, KEMRI-University of Oxford Wellcome Trust Collaborative Programme, Malaria Public Health and Epidemiology Group, Centre for Geographic Medicine, Nairobi, Kenya; 3 Human Development and Health Academic Unit, Southampton General Hospital, University of Southampton, Southampton, UK

**Keywords:** Bibliometrics, Citations, Impact factor, Systematic reviews

## Abstract

**Background:**

Systematic reviews are important for informing clinical practice and health policy. The aim of this study was to examine the bibliometrics of systematic reviews and to determine the amount of variance in citations predicted by the journal impact factor (JIF) alone and combined with several other characteristics.

**Methods:**

We conducted a bibliometric analysis of 1,261 systematic reviews published in 2008 and the citations to them in the Scopus database from 2008 to June 2012. Potential predictors of the citation impact of the reviews were examined using descriptive, univariate and multiple regression analysis.

**Results:**

The mean number of citations per review over four years was 26.5 (SD ±29.9) or 6.6 citations per review per year. The mean JIF of the journals in which the reviews were published was 4.3 (SD ±4.2). We found that 17% of the reviews accounted for 50% of the total citations and 1.6% of the reviews were not cited. The number of authors was correlated with the number of citations (*r* = 0.215, *P* < 0.001). Higher numbers of citations were associated with the following characteristics: first author from the United States (36.5 citations), an ICD-10 chapter heading of *Neoplasms* (31.8 citations), type of intervention classified as *Investigation*, *Diagnostics* or *Screening* (34.7 citations) and having an international collaboration (32.1 citations). The JIF alone explained more than half of the variation in citations (*R*^2^ = 0.59) in univariate analysis. Adjusting for both JIF and type of intervention increased the *R*^2^ value to 0.81. Fourteen percent of reviews published in the top quartile of JIFs (≥ 5.16) received citations in the bottom quartile (eight or fewer), whereas 9% of reviews published in the lowest JIF quartile (≤ 2.06) received citations in the top quartile (34 or more). Six percent of reviews in journals with no JIF were also in the first quartile of citations.

**Conclusions:**

The JIF predicted over half of the variation in citations to the systematic reviews. However, the distribution of citations was markedly skewed. Some reviews in journals with low JIFs were well-cited and others in higher JIF journals received relatively few citations; hence the JIF did not accurately represent the number of citations to individual systematic reviews.

## Background

Systematic reviews can guide clinical practice and health policy. The number of systematic reviews published in the literature is increasing at a steady rate. It was estimated that in 1990 there were approximately 250 published systematic reviews on healthcare [1]. In August 2013, the *Cochrane Database of Systematic Reviews* contained 5,637 reviews and 2,405 protocols, and the Database of Abstracts of Reviews of Effectiveness (DARE) contained over 24,000 reviews. In the United Kingdom, there are now evidence synthesis teams based in academic institutions which specialise in undertaking systematic reviews for national bodies such as the National Institute for Health and Care Excellence (NICE), the National Institute for Health Research Health Technology Assessment (NIHR HTA) Programme and other funders. Similarly, in the United States, the Agency for Healthcare Research & Quality (AHRQ) Technology Assessments Program commissions reviews based on a systematic review of the literature by a group of research teams in the United States and Canada. Thus, systematic reviews are an essential component of the HTAs that underpin policy decisions, and the increasing use of HTAs by policymakers has been one of the drivers of the increasing number of systematic reviews.

However, producers of systematic reviews in academic institutions need to justify performing them in terms of academic performance measures, such as publications and citations. These measures are important when competing for research funds and also for professional status and career progression, as well as in the recruitment of new staff. Performance is based partly on the number of publications in peer-reviewed journals and partly on the impact of those publications, as reflected in citation rates. In addition, citation rates may be used by funders of research as one indicator of the impact and dissemination of research they have funded. Research-active institutions will therefore wish to maximise citation rates to increase their success in securing funding.

### Journal impact measures

The journal impact factor (JIF) is obtained from the *Journal Citation Reports* (Thomson Reuters, New York, NY, USA) and is a measure of journal prestige and impact [2]. The impact factor is calculated by dividing the number of citations in the year by the total number of articles published in the two previous years. For example, the 2010 impact factor equals the number of citations in 2010 to items published in 2008 and 2009 divided by the number of items published in 2008 and 2009. In 2007, the five-year JIF was introduced. It is similar in nature to the two-year impact factor, but citations in a given year are counted back to the previous five years and divided by the number of source items published in the previous five years. It was thought that a base of five years might be more appropriate for journals in certain fields, where the body of citations may not be large enough to make reasonable comparisons, or that it might take longer than two years to disseminate and respond to published works.

Other journal metrics have come into use more recently. The SCImago Journal & Country Rank (SJR) uses a three-year citation window. The choice of three years as the publication window (rather than two or five years used for JIFs) is based on the observation that citations in many fields have not peaked after two years and citations in other fields have peaked too early for a five year cut-off [3]. The SJR also differs from JIFs in that not all citations are counted as being equal; that is, it weights the citations received according to the prestige of the citing journal [4]. Another journal metric is the Source Normalized Impact per Paper, or SNIP. It measures contextual citation impact by weighting citations on the basis of the total number of citations in a subject field, hence correcting for differences in citation potential and topicality between subject fields [3,5].

However, the most widely used and known of the journal metrics is the two-year JIF. Journal editors strive to improve their journal’s impact factor, as it is key to the journal’s ability to attract the best papers and hence to the survival of the journal [6-8].

Although it is intended to rate journals, the JIF of the journal in which an article has been published is widely used by academics and funding bodies as a surrogate measure of the quality and impact of the article itself [9-11], and some universities will instruct researchers to publish only in journals with an impact factor above a certain level [7]. However, as the distribution of citations to individual articles in a journal is known to be skewed and is often driven by a few highly cited articles, the JIF does not accurately reflect citations to the average article in the journal [12-15].

In some universities in the United Kingdom, JIFs will act as an important determinant in the selection of research papers (academic “outputs”) for the UK Research Excellence Framework (REF) in 2014 [15]. There is a common assumption that publication in journals with high JIFs will be associated with higher numbers of citations. Therefore, knowing how well JIFs, as well as other factors, predict citations to systematic reviews may be useful to those undertaking or planning systematic reviews in academic institutions preparing for the REF or similar academic assessment exercises in other countries and could be useful for formulating a publications strategy that will maximise the citation rates for systematic reviews.

Therefore, our primary aim was to undertake a bibliometric analysis of systematic reviews and to determine how well the JIF, alone and in combination with several other characteristics, predicts citations to systematic reviews. Our secondary aim was to determine the characteristics associated with systematic reviews that distinguish those that are highly cited from those that receive few or no citations.

## Methods

### Search strategy for systematic reviews

#### Terminology

Many, but not all, systematic reviews contain meta-analyses. In some cases, it is not possible or valid to perform a meta-analysis of the included studies because of clinical, methodological or statistical differences between studies [16]. Therefore, we will use the term *systematic reviews* collectively to refer to both systematic reviews that include a meta-analysis and those that do not.

#### Searches of the Scopus database

We searched the Scopus database in June 2012 using the following search strategy: “meta-analysis or systematic review” in the Title field only, limited to Document Type = Review, publication year = 2008, Subject Areas = Life Sciences or Health Sciences and Language = English language. The Preferred Reporting Items for Systematic Reviews and Meta-Analyses (PRISMA) Statement recommends that all systematic reviews or meta-analyses describe themselves with either or both of the words *meta-analysis* and *systematic review* in the title [17]. This search identified 1,381 articles and the bibliographic details and number of citations to each review were exported into Reference Manager.

### *Searches of the* Cochrane Database of Systematic Reviews

We performed a separate search of the Cochrane Database to identify Cochrane reviews to include in our data set, as Cochrane reviews are not described as systematic reviews in the title. Also, as they are regularly updated, both earlier and later versions of the same review may be cited; therefore, it was necessary to check each review to determine whether it was the current version and first published in 2008. We searched the *Cochrane Library*, issue 6, of 12 June 2012, limiting the search to 2008, and thus identified 152 reviews. The full text of each review was downloaded, and the history section of the review was checked to determine whether the review was first published in 2008. Methodology reviews and reviews that had been withdrawn were excluded. This led us to identify 79 reviews which were new to the Cochrane Database in 2008 that represented the current version. We then searched for these reviews in Scopus, and we downloaded the bibliographic details and number of citations into Reference Manager and Microsoft Excel (Microsoft, Redmond, WA, USA) for analysis.

The number of records gathered from both searches in Reference Manager was 1,460, and the abstracts were all screened for inclusion. To meet the inclusion criteria on the basis of the abstract, the review had to appear to have a clearly focused aim and an adequate search strategy, and it had to report the inclusion criteria. These criteria were based on those for the DARE database [18]. The abstracts of each review were checked, and we excluded articles that were not systematic reviews or did not include human studies and those that were methodological reviews or reviews of reviews. The full text was obtained for 95 studies in which the eligibility criteria could not be determined from the abstract. If the article did not include the criteria mentioned above or have a table of the characteristics of the included studies, it was excluded. A further 199 records were removed, which left 1,261 systematic reviews remaining in the data set. Additional file 1: Figure S1 shows the flow diagram for the searches.

The searches were performed in June 2012, so this gave an average time of four years to accumulate citations (with a range from 3.5 to 4.5 years). We considered four years to be enough time to accumulate sufficient citations to show differences between reviews.

#### Obtaining data for the characteristics of the systematic reviews

We collected data on the following variables for each systematic review: (1) JIF; (2) JIF −5 years; (3) number of pages of the review; (4) country location of the authors; (5) number of authors; (6) international collaboration; (7) condition or disease classified by the *International Statistical Classification of Diseases and Related Health Problems, 10th Revision* (ICD-10) [19], chapter code; and (8) type of intervention (for example, drug, nonpharmacological treatment, investigation). Variables 1 and 2 are characteristics of the journal in which the review was published, and variables 3 to 8 are characteristics unique to the article. Data on the number of citations, number of authors, country location of authors and number of pages per article were extracted from the information exported from bibliographic data in the Scopus database.

#### Impact factors

The two-year and five-year JIFs for each journal title were obtained manually from the *Journal Citation Reports: JCR Science Edition 2010* impact factors, published by Thomson Reuters (hereinafter *JIF* will refer to the impact factor measured over two years, and JIF-5 will refer to the five-year impact factor).

#### SCImago Journal & Country Rank

The 2011 SJR data were downloaded into an Excel file from the SCImago Journal & Country Rank website, which gave a complete list of journal rankings [20]. These data were imported into Microsoft Access and matched via the journal titles field in our data set.

#### Number of pages

The number of pages of each article was obtained from Scopus and was based on the start page and the end page of each review. For reviews in which this information was not given (such as in electronic journals), the review was downloaded and the pages of the main article were manually counted. This count did not include the pages in the supplementary data or appendices available online only.

#### ICD-10 chapter code

The coding of topics was carried out using the 22 codes in ICD-10 version 2010 [19], plus an additional code = 99 for ‘Uncertain or not known’. Each abstract was read by one author (NW) and classified into one of the 22 disease codes. These were checked by a second author (PR), and any differences were resolved by discussion.

#### Coding of type of intervention

The classification of each type of intervention was devised by one author (NW). It comprised the following 12 intervention types: (1) drugs; (2) surgery (including operations, fixation of fractures by operation of immobilisation); (3) health promotion; (4) investigations, diagnostics or screening; (5) psychological therapies; (6) vaccines; (7) alternative therapies (such as acupuncture, homeopathy, herbal medicines); (8) dentistry (covering “operation” and application of drugs such as fluoride gels or fissure sealants); (9) not an intervention; (10) mixed (some reviews cover all possible treatments, such as drugs, surgery and acupuncture); (11) vitamins, food supplements, exclusion diets and foods; and (12) Other.

As many systematic reviews are now on topics that are not interventions, a category of “Not an intervention” was necessary. Although systematic reviews have often been associated with interventions, this is now changing, as exemplified by the Cochrane Database, which initially included only reviews of treatments but more recently has included reviews of diagnostic methods.

Each review was classified by one author (NW) on the basis of the abstract into one of the 12 intervention types. These were checked by a second author (PR), and any differences were resolved by discussion.

#### Country location of the authors and international collaboration

The full institutional address of each author was exported from the Scopus database into Excel files, and this was used to determine the country location of the first author and all coauthors. Any articles that included authors with addresses from different countries were coded as international collaborations.

### Statistical analysis

Data were imported into SPSS version 20 software (SPSS, Chicago, IL, USA) and Stata version 12 software (StataCorp, College Station, TX, USA) from Microsoft Excel files. Descriptive analyses and Pearson correlations for continuous variables were performed in SPSS and Stata. In the univariate analysis for categorical variables, the Kruskal-Wallis test was used in SPSS to test the statistical significance of any differences in the categories with respect to the non–normally distributed continuous variable, citations.

The number of citations (the dependent variable) was positively skewed; therefore, the natural log transformation was obtained to approach a normal distribution. As some reviews had zero citations, the number 1 was first added to the number of citations to overcome the problem of log transformation of zero values.

Some of the predictor (independent) categorical variables (country location of first author, ICD-10 chapters and intervention type) had a large number of categories, which resulted in small numbers in some categories. Therefore, some categories were combined, and dummy variables were created as reference categories for regression analysis.

We present the *R*^2^ values, which represent the amount of variance contributed by each variable in the different models, to explain the citations in the results of the multivariate linear regression model rather than presenting the regression coefficients and associated 95% confidence intervals of log-transformed citations. A *P*-value ≤ 0.05 was considered statistically significant. All multivariate analyses were conducted using Stata version 12 software.

## Results

### Distribution of citations

The search of Scopus resulted in 1,261 systematic reviews published in 2008. The number of citations varied from zero to 221, and the reviews were published in 613 different journals. The four journal titles which accounted for the highest number of reviews were the *Cochrane Database of Systematic Reviews* (*n* = 79; 6.3%), *Health Technology Assessment* (*n* = 21; 1.7%), *Annals of Internal Medicine* (*n* = 18; 1.4%) and *JAMA* (*n* = 12; 1.0%). The remaining reviews were widely scattered, and 379 journals contained only one review.

The citations to the reviews were heavily skewed: 5.5% of the top-cited reviews accounted for 25% of the total citations, and 17% of the reviews accounted for 50% of the total citations. Also, 50% of the reviews contributed 84% of the total citations. Twenty reviews (1.6%) were not cited.

The characteristics of the 1,261 systematic reviews are shown in Table 1. The mean number of citations per review, accumulated after a mean of four years, was 26.5 (SD ±28.9). This equated to a mean of 6.6 citations per review per year.

**Table 1:** **Characteristics of systematic reviews (*N *= 1,261) published in 2008**^**a**^

**Characteristics**	**Data**
Citations over a mean of four years, mean (±SD)	26.5 (28.9)
Two-year JIF, mean (±SD; *n* = 1,101)	4.3 (4.2)
Five-year JIF (mean ± SD; *n* = 1,016)	4.6 (4.1)
Number of authors, mean (±SD)	4.3 (2.7)
Number of pages, mean (±SD)	16.0 (25.6)
Country location of first author, *n* (%)	
United Kingdom	301 (23.9)
United States	285 (22.6)
Canada	145 (11.5)
The Netherlands	83 (6.6)
Australia	83 (6.6)
All other countries	364 (28.9)
ICD-10 chapters, *n* (%)	
Neoplasms	135 (10.7)
Diseases of the circulatory system	120 (9.5)
Factors influencing health status and contact with health services	119 (9.4)
Mental and behavioural disorders	103 (8.2)
Diseases of the digestive system	103 (8.2)
All other ICD-10 codes	681 (54.0)
Intervention type, *n* (%)	
Not an intervention	443 (35.1)
Drugs and vaccines	243 (19.3)
Surgery and dentistry	139 (11.0)
Investigations, diagnostics or screening	128 (10.2)
Health promotion	45 (3.6)
All other interventions	263 (20.9)
International collaboration, *n* (%)	
No	1,057 (83.8)
Yes	204 (16.2)

^a^ICD-10, *International Statistical Classification of Diseases and Related Health Problems, 10th Revision*; JIF, journal impact factor.

Eighty-seven percent of the reviews were published in a journal with a JIF, and the mean JIF was 4.3 (SD ±4.2). Also, 80.6% of the reviews were in journals with a five-year JIF, and the mean JIF-5 was 4.6 (SD ±4.1).

The JIFs and the number of citations were both divided into quartiles, and a comparison was made between the top JIF quartile that had citation numbers in the bottom quartile and vice versa. There were 1,101 reviews published in journals with a JIF. We found that 14% (38 of 275) of reviews in the top JIF quartile (≥ 5.16) received citations in the bottom quartile (≤ 8 citations), and 9% (25 of 275) of reviews in the bottom JIF quartile (≤ 2.06) received citations in the top quartile (≥ 34 citations). Also, 6.3% (10 of 160) of reviews with no JIF had citation numbers in the top quartile.

### Characteristics of the systematic reviews

The mean number of authors per review was 4.3 (SD ±2.7), and the mean number of pages per review was 16 (SD ±25.6), but the latter did not take into account the pages in the supplementary data or appendices available online only in some print journals.

As the categorical variables, country locations of first author, ICD-10 chapters and intervention types all had a large number of categories, some were combined to derive sufficient numbers in each category. The numbers in each category, before being combined, are given in Additional File 2.

Table 1 shows that the United Kingdom had the highest percentage (24%) of first authors, followed by the United States (23%) and Canada (12%). The ICD-10 chapter with the highest number of reviews was ‘Neoplasms’ (10.7%), followed by ‘Diseases of the circulatory system’ (9.5%) and ‘Factors influencing health status and contact with health services’ (9.4%).

Examination of the intervention types shows that the highest percentage (35%) of the reviews was in the category ‘Not an intervention’. These reviews were on a very wide range of topics. The commonest were epidemiological reviews, such as the incidence or cause of diseases (for example, the role of risk factors in cardiovascular disease). These were followed by reviews of factors affecting use of healthcare (for example, ethnic variations in uptake), reviews of outcomes of care (for example, trends over time in survival) and economic reviews including quality-of-life results and ‘burden of disease’. The next most common type of intervention reviews was in the category ‘Drugs and vaccines’ (19%). Sixteen percent of reviews were international collaborations, and all authors had addresses within the same country in the remaining eighty-four percent.

### Continuous variables and citations

Table 2 explores the correlation between citations and the five continuous variables measured.

**Table 2:** Correlation between continuous variables and citations to systematic reviews

**Characteristics**	**Correlation with citations**	***P*-value**
Journal impact factor (JIF)	0.453	0.000
Journal impact factor-5 (JIF-5)	0.444	0.000
SCImago Journal Rank (SJR)	0.438	0.000
Number of authors	0.215	0.000
Number of pages	−0.002	0.943

Four of the variables (JIF, JIF-5, SJR and number of authors) were highly correlated (*P* < 0.000) with the number of citations. The highest correlation (0.453) was with the JIF. The number of pages was not significantly associated with the number of citations (*P* = 0.943).

### Categorical variables

Table 3 shows the mean number of citations accumulated over four years for the categorical variables after combining some categories with low numbers of citations. The mean number of citations for all variables in each category, prior to combining categories, is given in Additional File 2.

**Table 3:** **Categorical variables predicting citations to systematic reviews (mean number of citations and adjusted odds ratios)**^**a**^

**Variables**	**Mean number of citations (±SD)**	***P*-value**
Country location of first author		
United States	36.5 (37.4)	0.000
The Netherlands	29.0 (34.3)	
Canada	24.4 (24.6)	
United Kingdom	23.8 (25.9)	
Australia	23.0 (19.5)	
All other countries (reference category)	22.1 (23.4)	
ICD-10 chapters		
Neoplasms	31.8 (30.9)	0.000
Mental and behavioural disorders	29.5 (27.7)	
Diseases of the circulatory system	29.1 (31.5)	
Factors influencing health status and contact with health services	26.0 (31.1)	
Diseases of the digestive system	17.0 (18.7)	
All other ICD-10 codes (reference category)	26.2 (28.8)	
Intervention type		
Investigations, diagnostics or screening	34.7 (34.8)	0.009
Drugs and vaccines	27.8 (30.4)	
Not an intervention	26.9 (29.2)	
Health promotion	25.2 (25.3)	
Surgery and dentistry	21.7 (22.2)	
Other treatments (reference category)	23.6 (26.8)	
International collaboration		
Yes	32.1 (34.3)	0.000
No (reference category)	25.5 (27.6)	

^a^ICD-10, *International Statistical Classification of Diseases and Related Health Problems, 10th Revision*.

### Country location of first author

There was a significant difference in the mean number of citations between the country locations of the first authors (*P* < 0.000). The highest (36.5) was for reviews with U.S. first authors, followed by those from the Netherlands (29.0).

### ICD-10 chapters

A significant difference (*P* < 0.000) was found in the mean number of citations to reviews with respect to the ICD-10 chapter codes. The highest mean number of citations (31.8) was for the chapter heading ‘Neoplasms’. This was followed by ‘Mental and behavioural disorders’ and ‘Diseases of the circulatory system’, with means of 29.5 and 29.1 citations, respectively.

### Type of intervention

There was a significant difference (*P* = 0.009) in the mean number of citations between the different types of interventions. The intervention type with the highest mean number of citations (34.7) was ‘Investigations, diagnostics or screening’, followed by ‘Drugs and vaccines’ (27.8).

### International collaboration

The mean number of citations to reviews which had authors from more than one country (32.1) was significantly higher (*P* = 0.000) than for those where all authors were from the same country (25.5).

### Regression analysis

We performed multiple regression to determine the amount of variation in citations explained by the JIF and the additional amount explained by each of the five variables added to JIF. The results given in Table 4 show that the JIF *R*^2^ = 0.592; that is, the JIF alone accounted for 59.2% of the variation in citations of reviews. The variable, which, when added to the JIF, explained the most variation was the intervention type, which explained an additional 21.4%. The additional variations explained individually by each of the other factors were country location of first author (17.1%), number of authors (16.7%), ICD-10 code (10%) and international collaboration (2.7%).

**Table 4:** ***R***^**2 **^**values after adjustment in multiple regression analysis**^**a**^

**Factors adjusted for in multiple regression**	***R***^**2 **^**values**
Journal impact factor	0.592
Journal impact factor and intervention type	0.806
Journal impact factor and country location of first author	0.763
Journal impact factor and number of authors	0.759
Journal impact factor and ICD-10 code	0.692
Journal impact factor and international collaboration	0.619

^a^ICD-10, *International Statistical Classification of Diseases and Related Health Problems, 10th Revision*.

### Characteristics of the journals with the top and bottom 50 number of citations

We compared reviews that had the top 50 and bottom 50 numbers of citations. The 50 most-cited reviews were spread over 32 different journals. The number of citations ranged from 92 to 221. The *Annals of Internal Medicine* had eight reviews, *JAMA* had six reviews and six other journals contained two reviews each. The remaining 24 journals contributed just one review each. The *Cochrane Database of Systematic Reviews* and *Health Technology Assessment*, the publishers of the most reviews in this study, contained one and zero reviews, respectively, that were in the top 50 cited. The 50 least-cited reviews were spread over 45 different journals. The number of citations ranged from zero to two. Three reviews were from the *Cochrane Database of Systematic Reviews*.

Table 5 shows the characteristics of the 50 most-cited and the 50 least-cited systematic reviews. There is a statistically significant difference in the mean number of citations, JIFs and number of authors, and in the percentages of international collaboration, reviews published in journals indexed in MEDLINE and journals with a JIF. There was no statistically significant difference with regard to the number of pages of the review.

**Table 5:** **Comparison of the 50 most-cited versus 50 least-cited reviews**^**a**^

**Characteristics**	**Top 50 cited**	**Bottom 50 cited**	***P*-value of difference**
Number of citations (mean)	132.0 (SD ±29.7)	0.6 (SD ±0.5)	<0.000
JIF (mean)	10.5 (SD ±8.89)	2.3 (SD ±2.3)	<0.000
Number of authors (mean)	6.3 (SD ±6.4)	3.5 (SD ±2.2)	0.004
Number of pages (mean)	12.5 (SD ±9.4)	13.7 (SD ±27.9)	0.790
International collaboration	24%	8%	0.029
Published in journals with JIF	100%	50%	<0.000
Indexed in MEDLINE	100%	54%	<0.000
Top ICD-10 chapter	16% ‘Neoplasms’	20% ‘Diseases of the respiratory system’	
Top intervention type	40% ‘Not an intervention’	28% ‘Not an intervention’	
Top country location of first author	48% United States	34% United Kingdom	

^a^ICD-10, *International Statistical Classification of Diseases and Related Health Problems, 10th Revision*; JIF, journal impact factor.

The most common ICD-10 chapter in the top 50 was ‘Neoplasms’ , and ‘Diseases of the respiratory system’ was the most common in the bottom 50. The most common country location of first authors in the top 50 was the United States, and the United Kingdom was most the common first-author country location in the bottom 50 cited.

## Discussion

In this study, we examined several characteristics of systematic reviews and citations to them four years after publication. The citations to the reviews were heavily skewed, with 17% of the reviews accounting for 50% of the total citations. Also, 14% of reviews that were published in journals in the top quartile of JIFs received citations in the bottom quartile, 9% of reviews published in journals in the lowest JIF quartile received citations in the top quartile and 6% of reviews in journals with no JIF were also in the top quartile of citations.

The univariate analysis showed that adjusting for JIF alone showed it predicted 59% of the citations. When the data were adjusted for both the JIF and type of intervention, the *R*^2^ value increased to 0.81, so these two factors explained 81% of the variance in the citations.

An examination of the top 50 versus bottom 50 reviews cited showed that the journals *Annals of Internal Medicine* and *JAMA* contained the highest number of highly cited reviews. The *Cochrane Database of Systematic Reviews* published only one review in the top 50, despite being the journal with the most reviews in the total data set.

### Strengths and limitations of this study

The main strengths of this study are that it is the first to look at predictors of citations specifically to systematic reviews published over a wide range of subject areas and journals, and it included a number of characteristics of both the article itself and the journal in which it appeared. Possible limitations included the fact that not all systematic reviews would be captured in our search. We restricted our search to the English-language literature and to reviews with the words *systematic review* or *meta-analysis* in the title (as recommended in the PRISMA statement). Therefore, it is possible that such reviews are of higher quality and hence receive more citations than other systematic reviews.

### Other studies and models used to predict citations to articles in medical journals

Other studies have looked at predictors of citations to articles in medical journals. Lokker and colleagues investigated whether citation counts at two years could be predicted for clinical articles that pass basic critical appraisal criteria data available within three weeks of publication [21]. They collected 20 variables for each article, and included 1,261 articles published in 105 journals. Cochrane reviews and articles from the HTA database accounted for 24% of the sample. Their results showed that the regression equation accounted for 60% of the variation in citations (*R*^2^ = 0.60, 95% CI = 0.54 to 0.63; *P* < 0.001). Eleven variables remained statistically significant in their regression model. However, most of the variables collected in their study differed from those in this study (except for number of pages and number of authors) and did not include the JIF. Also, they included only articles that met specific quality criteria and did not limit articles to systematic reviews only.

Kulkarni and colleagues examined features of articles associated with higher citation rates in original articles, regardless of study methodology, published in three general medicine journals with high impact factors [22]. They extracted data on nine variables from three hundred twenty-eight articles and analysed them for their association with the annual rate of citations per article five years after publication. The following variables were retained in a multivariable regression model: industry funding, industry-favouring result, clinical category of article, group authorship, journal of publication and sample size. The model explained approximately 20% of the variance (adjusted *R*^2^ = 0.20) in annual citation rates of the cohort of articles. As the authors mentioned, however, these results are not generalizable to articles published in periodicals other than the three high-impact general medical journals they reviewed. In contrast to our present study, Kulkarni and colleagues included all original articles of any methodology and did not adjust for JIF in their model.

Callaham and colleagues identified characteristics predicting citations for a standardized 3.5 years after publication to 204 published articles originally submitted as abstracts to a 1991 emergency medicine meeting [23]. The ability to predict the citations per year was weak (pseudo-*R*^2^ = 0.14). Of the 11 variables included in the regression model, the strongest predictor of citations was the JIF of the publishing journal. After adjustment for the JIF, the presence of a control group, the subjective newsworthiness score and the sample size were the next most important determinants of citation. They found no relationship between study design (and other measures of quality) and JIF. Although Callaham and colleagues included JIF as a predictor variable, they did not include any other variables similar to those included in our present study. Also, in their study, they looked only at research in one subject area and arising from only one specialty meeting, and they included all study designs.

### Distribution of citations

In this study, we found that just 17% of the reviews accumulated 50% of the total citations and that 14% of reviews in the journals with the higher JIFs were in the bottom quartile of citations. Conversely, 15% of reviews in the bottom JIF quartile or with no JIF were in the top quartile of citations. This skewed distribution of citations to systematic reviews is consistent with that reported in other studies in medical journals [13,14,24]. Falagas and colleagues looked at the distribution of citations in clinical medicine journals for original research articles and review articles in high-, moderate- and low-impact journals and found that 12% to 18% of review articles accounted for 50% of the citations, and this percentage did not vary markedly between journals of different JIF levels [14]. Therefore, articles published in a low-JIF journal can still be oft-cited, and, conversely, articles appearing in high-JIF journals can receive few or no citations.

### Length of reviews and citations

Lokker and colleagues found a statistically significant negative association between citation count and article length, but this association disappeared when Cochrane reviews and HTA reports were removed from the analysis [21]. A positive relationship between article length and citation count was reported by Falagas and colleagues, but they looked at articles in only five general medical journals, with a maximum length of 15 pages and with reviews excluded [14].

We originally expected that longer reviews might be wider in scope, more complex and of higher methodological quality (owing to more included studies, detailed reporting of the quality assessment and study characteristics, more sensitivity analyses in the results and a more thorough discussion) and hence might receive more citations. However, we found that the length of reviews was not significantly associated with the number of citations.

We hypothesise that the relationship between citation count and number of pages may be different in systematic reviews. Some HTA reports and Cochrane reviews in this study were over 200 pages long and contained long Methods sections and data extraction tables, which many readers may skip over. Such length might deter people from printing, reading and citing them, as many people still prefer to print out articles rather than read them on their screens.

### Higher citation rates of systematic reviews

Our study seems to confirm the view that study designs with higher methodological rigour, such as systematic reviews and meta-analyses, have a higher citation rate than other study designs [25-31]. The systematic reviews included in this study had a mean of 26.5 citations over four years, which gave a mean of 6.6 citations per review per year, whereas the mean two-year and five-year JIFs of the journals in which they were published were 4.3 and 4.6, respectively. These data indicate that, overall, systematic reviews perform above average for the journals in which they appear and therefore may increase the JIFs of the journals in which they are published.

### Uncitedness

In this study, we found that only 20 reviews (1.6%) remained uncited after four years. The lower rates of noncitation of reviews was also found by Weale and colleagues, who looked at total citations gained by October 2003 for every original article and review published in immunology and surgery during 2001 [24]. Of the 30,208 articles, 24.3% were uncited by October 2003. The level of noncitation was significantly lower for reviews (14.8%) than for original articles (24.9%) (*P* < 0.0001).

### Impact of number of authors and international collaboration

We observed that both international collaboration and the number of authors improve citations. This could be explained by the fact that having international collaboration and a large number of authors may reflect the complexity of the topic and hence the range of skills required to do the review and perhaps the importance of the topic. Figg and colleagues also observed that the number of times an article is cited is significantly and positively related to the number of authors and institutions [32].

We speculate that another explanation for the association of higher citations with number of authors and international collaboration may be information gain. This was described by Evangelou and colleagues, who found that reviews that substantially reduce uncertainty may be particularly highly cited [33]. They looked at the correlation between the information gain from randomized trials and their publication in high-JIF journals and quantified how much the new findings changed established knowledge. They found that publication in journals with high JIFs is driven by how extensively the results of a study change prior perceptions of the evidence, independently of the statistical significance of the results and the size of the trial and extent of heterogeneity of the meta-analysis results.

### Influence of subject area and type of intervention on citations

The ICD-10 code ‘Neoplasms’ had the highest mean number of citations in our present study. Kulkarni and colleagues analysed features associated with higher citation rates in original articles published in four high-JIF general medicine journals, regardless of study methodology [22]. In their adjusted analysis, higher annual rates of citation were also associated with articles dealing with cardiovascular medicine (13.3 more) and oncology (12.6 more).

The higher citations of reviews of the intervention types classified as ‘Investigations, diagnostics or screening’ may reflect the need, in a time of limited resources, to look critically at interventions other than drugs, especially because many drugs have already been reviewed. Indeed, studies of some drugs may have been reviewed several times. Siontis and colleagues recently reported that overlapping meta-analyses on the same topic were common, and all of their examples of “multiple meta-analyses” were on medication-related topics [34]. Higher citations to diagnostics reviews may also reflect the development in the methods used for evaluating diagnostic technologies, such as in the Cochrane Collaboration. In the United Kingdom, NICE, best known for issuing guidance on new drugs, has started a diagnostics assessment programme.

### Why are Cochrane reviews not more frequently cited?

We observed that only one review from the *Cochrane Database of Systematic Reviews* was in the top 50 cited, but three were in the bottom 50. This was surprising, given that free or funded free access to the Cochrane Library is widely available in many countries (but not in North America), and Cochrane reviews have been shown to be of higher quality than other reviews [35,36]. A study by McKinlay and colleagues, however, showed that even when access was provided equally to Cochrane and journal reviews, the former were less popular [37]. Some criticisms that have been levelled at Cochrane reviews that may explain this fact are that there are too many empty reviews (reviews in which only one or no randomised controlled trial are found), they lack relevance to clinical practice because of the very narrow focus of the questions, their length and complexity make them difficult to read and extract the key clinical messages, and they often lack a clear answer as to which treatment was better [38-41]. Also, as one of our referees suggested, another reason for low citations to some Cochrane reviews may be that the choice of topic is made by the reviewers and that the topics chosen may not be regarded as high priority by clinicians in that specialty.

### Unanswered questions and future research

There are other characteristics of systematic reviews not included in our model, which may also be predictors of citation rates, such as the quality of the review, whether the review included a meta-analysis, the number of studies included, the study design of the included articles, whether the review was positive or negative, whether the review included an economic evaluation, the number of existing reviews already done on the topic and the perceived information gain.

It would be interesting to investigate whether open access publications versus publication in a subscription-only journal increases citations. Because of the variety in open access provision (some journals are immediately open access, other journals allow open access to some articles and others allow delayed open access after an embargo period), however, it would be difficult to determine the access status of the review at the time of citation. Also of interest would be a study that investigates the difference in citation rates between reviews published in dedicated review journals and more general journals.

## Conclusions

Although JIFs were found to predict over half of the citations of the systematic reviews, the distribution of citations to them was markedly skewed. Some of the most highly cited reviews were in journals with the lowest JIFs, and some reviews in high JIF journals were poorly cited. Hence the JIF is not an appropriate surrogate measure of the impact of individual systematic reviews.

## Abbreviations

AHRQ: Agency for Healthcare Research & Quality; DARE: Database of abstracts of reviews of effectiveness; HTA: Health Technology Assessment; ICD-10: *International Statistical Classification of Diseases and Related Health Problems, 10th Revision*; JIF: Journal impact factor; NICE: National Institute for Health and Care Excellence; NIHR: National Institute for Health Research; REF: Research Excellence Framework; SJR: SCImago Journal Rank; SNIP: Source normalized impact per paper.

## Competing interests

The authors declare that they have no competing interests.

## Authors’ contributions

PR designed the study, carried out the data extraction and analysis, and drafted the manuscript. NBK carried out the statistical analysis. KB carried out some of the data extraction. NW carried out some of the data extraction and drafted the manuscript. All authors contributed to and approved the final manuscript.

## Supplementary Material

Additional file 1 Flow diagram of searches for systematic reviews. Click here for file

Additional file 2 All categorical variables for Country of first author, ICD-10 Chapter and Intervention Type. Click here for file
