# Supplementary material for: Bibliometrics of systematic reviews: analysis of citation rates and journal impact factors
Source: Syst Rev. 2013 Sep 12;2:74. doi: 10.1186/2046-4053-2-74 (PMC3847500; doi:10.1186/2046-4053-2-74)
Supplement: Additional file 1 — Flow diagram of searches for systematic reviews. [file 2046-4053-2-74-S1.docx]

**Additional file 1** Flow diagram for identifying systematic reviews

Records identified through searching Cochrane Library
(n = 152)

Records identified through searching Scopus
(n = 1381)

Cochrane reviews excluded on basis of full text as not new in 2008
(n = 73)

Records remaining after combining searches

(n = 1460)

Records excluded on basis of abstract or full text as not being systematic reviews

(n=199)

Studies included in dataset

(n =1261)
