# Supplementary material for: Bibliometrics of systematic reviews: analysis of citation rates and journal impact factors
Source: Syst Rev. 2013 Sep 12;2:74. doi: 10.1186/2046-4053-2-74 (PMC3847500; doi:10.1186/2046-4053-2-74)
Supplement: Additional file 2 — All categorical variables for Country of first author, ICD-10 Chapter and Intervention Type. [file 2046-4053-2-74-S2.docx]

**Additional file 2 The number of systematic reviews and mean number of citations to all categorical variables**

1

**Country of first author**

| **Country of First Author -all countries** | **Number of**  **reviews** | **Mean (SD) Number of Citations** |
| --- | --- | --- |
| United Kingdom | 301 | 23.8 (25.9) |
| United States | 285 | 36.5 (37.4) |
| Canada | 145 | 24.4 (24.7) |
| Netherlands | 83 | 29.0 (34.4) |
| Australia | 83 | 23.0 (19.6) |
| Italy | 36 | 31.7 (26.1) |
| Germany | 36 | 24.4 (35.4) |
| Brazil | 34 | 13.1 (12.3) |
| China | 30 | 15.4 (14.0) |
| Greece | 26 | 26.1 (23.1) |
| Switzerland | 23 | 24.9 (27.4) |
| Spain | 20 | 23.7 (19.2) |
| Norway | 14 | 31.5 (32.1) |
| Denmark | 13 | 29.3 (24.7) |
| Finland | 12 | 18.8 (7.6) |
| Sweden | 11 | 15.0 (11.6) |
| Belgium | 10 | 53.2 (41.0) |
| France | 9 | 30.8 (26.6) |
| Iran | 8 | 18.5 (16.2) |
| New Zealand | 7 | 23.4 (21.0) |
| South Africa | 7 | 21.9 (15.0) |
| Japan | 7 | 10.9 (4.0) |
| Israel | 6 | 15.2 (9.6) |
| Ireland | 5 | 16.2 (18.0) |
| Taiwan | 5 | 11.2 (9.6) |
| Hong Kong | 5 | 10.8 (7.3) |
| Singapore | 4 | 9.8 (12.5) |
| Poland | 3 | 26.0 (32.2) |
| Nigeria | 3 | 8.7 (3.1) |
| South Korea | 2 | 17.0 (18.4) |
| Argentina | 2 | 15.5 (2.1) |
| India | 2 | 15.0 (12.7) |
| Russian Federation | 2 | 12.5 (5.0) |
| Thailand | 2 | 12.0 (11.3) |
| Bahrain | 2 | 8.5 (3.5) |
| Mexico | 2 | 4.5 (2.1) |
| Lithuania | 2 | 2.5 (2.1) |
| Uruguay | 1 | 39.0 |
| Egypt | 1 | 30.0 |

2

| Austria | 1 | 28.0 |
| --- | --- | --- |
| Uganda | 1 | 21.0 |
| Morocco | 1 | 21.0 |
| Tanzania | 1 | 19.0 |
| Philippines | 1 | 13.0 |
| Lebanon | 1 | 11.0 |
| Portugal | 1 | 8.0 |
| Kenya | 1 | 8.0 |
| Jamaica | 1 | 7.0 |
| Saudi Arabia | 1 | 6.0 |
| Korea | 1 | 3.0 |
| Colombia | 1 | 2.0 |
| **Total** | **1261** | **26.5** |

**ICD-10 Chapters**

| **ICD Chapters - all chapters** | **Number of**  **reviews** | **Mean (SD) Number of Citations** |
| --- | --- | --- |
| Neoplasms | 135 | 31.8 (30.9) |
| Diseases of the circulatory system | 120 | 29.1 (31.6) |
| Factors influencing health status and contact with health services | 119 | 26.0 (31.1) |
| Mental and behavioural disorders | 103 | 29.5 (27.7) |
| Diseases of the digestive system | 103 | 17.0 (18.7) |
| Diseases of the  musculoskeletal system and connective tissue | 99 | 23.6 (26.1) |
| Infectious and parasitic diseases | 87 | 30.8 (29.8) |
| Endocrine, nutritional and metabolic diseases | 76 | 38.4 (37.3) |
| Injury, poisoning and certain other consequences of external causes | 64 | 16.1 (14.7) |
| Diseases of the genitourinary system | 51 | 36.9 (44.7) |
| Unknown | 49 | 24.5 (23.4) |
| Pregnancy, childbirth and the puerperium | 48 | 20.5 (19.3) |
| Symptoms, signs and abnormal clinical and laboratory findings, not elsewhere classified | 41 | 20.4 (18.4) |
| Diseases of the nervous system | 40 | 21.8 (25.4) |

3

| Diseases of the respiratory system | 31 | 31.9 (36.6) |
| --- | --- | --- |
| Certain conditions  originating in the perinatal period | 18 | 23.2 (20.0) |
| Diseases of the blood and blood-forming organs and certain disorders involving the immune mechanism | 16 | 18.7 (16.6) |
| Diseases of the eye | 15 | 18.5 (14.6) |
| Congenital malformations, and chromosomal abnormalities | 10 | 19.3 (14.1) |
| Codes for special purposes | 10 | 17.0 (15.7) |
| Diseases of the skin and subcutaneous tissue | 9 | 42.4 (47.1) |
| External causes of morbidity and mortality | 9 | 30.6 (22.1) |
| Diseases of the ear and mastoid process | 8 | 9.0 (9.9) |
| **Total** | **1261** | **26.5** |

**Intervention type**

4

| **Intervention types - All Categories** | **Number of**  **reviews** | **Number of Citations** |
| --- | --- | --- |
| Not an intervention | 443 | 27.0 (29.2) |
| Drugs | 237 | 28.0 (30.6) |
| Investigations/diagnostics and screening | 128 | 34.7 (34.8) |
| Other treatments | 120 | 25.4 (29.3) |
| Surgery | 106 | 25.0 (23.6) |
| Alternative therapies | 66 | 14.4 (12.0) |
| Health promotion | 45 | 25.2 (25.3) |
| Dentistry | 33 | 10.8 (12.2) |
| Psychological therapies | 28 | 25.7 (23.0) |
| Mixed | 25 | 26.1 (34.0) |
| Vitamins, food  supplements, exclusion diets | 24 | 34.8 (33.6) |
| Vaccines | 6 | 17.8 (18.0) |
| **Total** | **1261** | **26.5** |
